# COVID-19**–**Associated Deaths After SARS-CoV-2 Infection During Pregnancy — Mississippi, March 1, 2020–October 6, 2021

**DOI:** 10.15585/mmwr.mm7047e2

**Published:** 2021-11-26

**Authors:** Laurin Kasehagen, Paul Byers, Kathryn Taylor, Theresa Kittle, Christine Roberts, Charlene Collier, Britney Rust, Jessica N. Ricaldi, Jamilla Green, Lauren B. Zapata, Jennifer Beauregard, Thomas Dobbs

**Affiliations:** ^1^Mississippi State Department of Health; ^2^Division of Reproductive Health, National Center for Chronic Disease Prevention and Health Promotion, CDC; ^3^University of Mississippi Medical Center, Jackson, Mississippi; ^4^CDC COVID-19 Response Team.

Pregnant and recently pregnant women are at increased risk for severe illness and death from COVID-19 compared with women who are not pregnant or were not recently pregnant (*1*,*2*). CDC recommends COVID-19 vaccination for women who are pregnant, recently pregnant, trying to become pregnant, or might become pregnant in the future.*^,^^†^ This report describes 15 COVID-19–associated deaths after infection with SARS-CoV-2 (the virus that causes COVID-19) during pregnancy in Mississippi during March 1, 2020–October 6, 2021.

The Mississippi State Department of Health (MSDH) identifies COVID-19 cases and deaths through required health care provider and hospital reporting^§^ and death certificate reviews. A COVID-19–associated death after SARS-CoV-2 infection during pregnancy was defined as the death of a woman with confirmed or probable SARS-CoV-2 infection during pregnancy who subsequently died during pregnancy or within 90 days after the pregnancy ended.^¶^ This study assessed characteristics of the decedents and timing of infection, for the periods before the highly transmissible SARS-CoV-2 B.1.617.2 (Delta) variant became predominant (March 2020–June 2021) and during Delta variant predominance (July 2021–October 2021).** For each period, the ratio of the number of COVID-19–associated deaths per 1,000 SARS-CoV-2 infections during pregnancy was assessed.^††^ Poisson 95% CIs were calculated using CDC's National Center for Health Statistics methods for computing confidence limits for a death rate based on a Poisson variable of 1–99 deaths.^§§^ This activity was reviewed by CDC and was conducted consistent with applicable federal law and CDC policy.^¶¶^

During March 1, 2020–October 6, 2021, a total of 1,637 SARS-CoV-2 infections during pregnancy were reported, and 15 COVID-19–associated deaths occurred (nine deaths per 1,000 SARS-CoV-2 infections). During the pre-Delta period, six COVID-19–associated deaths occurred (five deaths per 1,000 SARS-CoV-2 infections during pregnancy; 95% CI = 1.7–10.3); during the period of Delta predominance, nine COVID-19–associated deaths occurred (25 deaths per 1,000 SARS-CoV-2 infections during pregnancy; 95% CI = 11.3–46.8) (Table).

**TABLE:** Characteristics of women who died after SARS-CoV-2 infection during pregnancy before or during the period of SARS-CoV-2 B.1.617.2 (Delta) variant predominance — Mississippi, March 1, 2020–October 6, 2021

Characteristic	Total (Mar 1, 2020–Oct 6, 2021)	Before Delta predominance (Mar 2020–Jun 2021)	During Delta predominance (Jul–Oct 2021)
No. of COVID-19–associated deaths (deaths per 1,000 SARS-CoV-2 infections in pregnant women)*	**15 (9)**	6 (5)	9 (25)
**Age, median (range), yrs**	**30 (23–40)**	27 (23–38)	35 (23–40)
**Race/Ethnicity, no. (%)**			
Black, non-Hispanic	**9 (60)**	4 (67)	5 (56)
White, non-Hispanic	**3 (20)**	1 (17)	2 (22)
Hispanic	**3 (20)**	1 (17)	2 (22)
**Gestational age at symptom onset, median (range), wks**	**26 (8–37)**	34 (8–37)	24 (22–37)
**Gestational age at end of pregnancy or death, median (range), wks**	**28 (9–37)**	35 (9–37)	24 (22–37)
**Interval from symptom onset to death, median (range), days**	**18 (1–87)**	18 (1–87)	18 (9–45)
**Disease course/complications, no. (%)**			
Admitted to ICU	**15 (100)**	6 (100)	9 (100)
Invasive mechanical ventilation	**14 (93)**	5 (83)	9 (100)
Emergency cesarean delivery	**7 (47)**	1 (17)	6 (67)
Died during pregnancy	**3^†^ (20)**	1 (17)	2 (22)
Died after live birth	**12^§^ (80)**	5 (83)	7 (78)
**Underlying medical conditions, no. (%)**	**14 (93)**	6 (100)	8 (89)
Obesity	**10 (67)**	4 (67)	6 (67)
Hypertension^¶^	**8 (53)**	4 (67)	4 (44)
Diabetes (preexisting or gestational)	**4 (27)**	1 (17)	3 (33)
Cancer	**2 (13)**	0 (—)	2 (22)
HIV with pneumocystis pneumonia	**1 (7)**	1 (17)	0 (—)
**COVID-19 vaccination status, no. (%)**			
Fully vaccinated	**0 (—)**	0 (—)	0 (—)
Partially vaccinated	**1 (7)**	1 (17)	0 (—)
Unvaccinated**	**14 (93)**	5 (83)	9 (100)

**Abbreviation:** ICU = intensive care unit.

* The total number of SARS-CoV-2 infections in pregnant women was 1,637. The number of SARS-CoV-2 infections in pregnant women before and during Delta variant predominance was 1,272 and 365, respectively.

^†^ Three deaths during pregnancy resulted in one spontaneous abortion (9 weeks’ gestation) before Delta predominance and two stillbirths (22 and 23 weeks’ gestation) during Delta predominance.

^§^ Median = 5 days postpartum; range = 1–87 days.

^¶^ Before and during pregnancy.

** Five (83%) deaths in the period before Delta predominance occurred before vaccines were available.

The median age of the 15 decedents was 30 years (range = 23–40 years). Nine were non-Hispanic Black women, three were non-Hispanic White women, and three were Hispanic women. The median interval from symptom onset to death before and during Delta predominance was 18 days (pre-Delta range = 1–87 days; Delta range = 9–45 days). All decedents had been admitted to an intensive care unit, and 14 required invasive mechanical ventilation. Seven underwent emergency cesarean delivery (including two at the bedside). Three died during pregnancy, resulting in one spontaneous abortion at 9 weeks and two stillbirths at 22 and 23 weeks’ gestation, and 12 died after a live birth (median = 5 days postpartum, range = 1–87 days). Underlying medical conditions were present in 14 decedents. Receipt of monoclonal antibodies was not documented for any of the decedents. None of the 15 decedents had been fully vaccinated against COVID-19: five deaths occurred before COVID-19 vaccinations became available in December 2020; one decedent had been partially vaccinated; and nine were unvaccinated.

The findings in this report are subject to at least six limitations. First, there are limitations to identifying history of pregnancy from death certificates and through COVID-19 case reporting systems (*3*,*4*), which likely result in underascertainment of COVID-19 cases during pregnancy in Mississippi. Second, reported ratios of deaths per 1,000 SARS-CoV-2 infections during pregnancy might be overestimated if the total numbers of SARS-CoV-2 infections during pregnancy were undercounted. Third, because of the small number of deaths, the statistical significance of the difference in the ratios between periods was not assessed. Fourth, genomic sequencing was not performed on decedents’ viral samples for the deaths that occurred during July 2021–October 2021; however, the Delta variant accounted for nearly 100% of sequenced SARS-CoV-2 specimens in Mississippi during that period. Fifth, deaths among patients with more recent COVID-19 cases might be undercounted because less time has elapsed for the death to occur. Finally, an in-depth review of whether death was pregnancy-related (from any cause related to or aggravated by pregnancy) was not performed, so these data cannot be compared with pregnancy-related mortality ratios.*** Maternal mortality review committees (MMRCs)^†††^^,^^§§§^ identify all pregnancy-associated deaths as those occurring during pregnancy and ≤1 year after the end of pregnancy using linked death and birth certificate data.

This study found 15 COVID-19–associated deaths after SARS-CoV-2 infection during pregnancy (nine deaths per 1,000 SARS-CoV-2 infections); during the same period, 413 COVID-19–associated deaths were reported among females of reproductive age (2.5 deaths per 1,000 SARS-CoV-2 infections).^¶¶¶^ In addition, this study found an apparent increase in the ratio of COVID-19–associated deaths per 1,000 cases among pregnant women as the Delta variant became predominant (pre-Delta period: five deaths per 1,000 SARS-CoV-2 infections during pregnancy; Delta predominance period: 25 deaths per 1,000 SARS-CoV-2 infections during pregnancy). A similar increase in the ratio of deaths per 1,000 cases was observed for females of reproductive age in Mississippi, although the magnitude of the ratios was lower overall and by period (pre-Delta period: 2.1 deaths per 1,000 SARS-CoV-2 infections among females of reproductive age; Delta predominance period: 3.3 deaths per 1,000 SARS-CoV-2 infections among females of reproductive age). Twelve of the 15 decedents were Black women or Hispanic women. In comparison, during March 2020–October 2021 in Mississippi, an estimated 43% of births were among Black women and an estimated 5% of births were among Hispanic women. The Mississippi MMRC will conduct a comprehensive, multidisciplinary review of all pregnancy-associated deaths among Mississippi residents, including those attributable to COVID-19, to determine relatedness to pregnancy and contributing factors, including inequities in social determinants of health, and to develop recommendations for the prevention of future deaths. CDC recommends COVID-19 vaccination for pregnant women to prevent serious illness, death, and adverse pregnancy outcomes from COVID-19. Given existing disparities in vaccination rates among pregnant women,****^,^^††††^ partnerships to address vaccine access, hesitancy, or other concerns about vaccination can enhance fair and just access to COVID-19 vaccination, including among Black persons and Hispanic persons.

## Footnotes

*
https://emergency.cdc.gov/han/2021/han00453.asp

†
https://www.cdc.gov/coronavirus/2019-ncov/vaccines/recommendations/pregnancy.html#anchor_1628692562866

§ COVID-19 infection, including COVID-19–associated death, was added to the Mississippi List of Reportable Diseases and Conditions on March 10, 2020. Reported deaths are reviewed alongside surveillance data to ascertain the presence of a case report or a positive laboratory test result and to determine whether the death resulted from an acute SARS-CoV-2 infection. A review of each death established clinical characteristics and pregnancy status at time of infection.

¶ For COVID-19–associated deaths, pregnancy status at time of SARS-CoV-2 infection was determined based on direct communications from health care providers or hospitals, COVID-19 case report forms, or death certificates. For COVID-19–associated deaths after SARS-CoV-2 infection during pregnancy identified through this process, pregnancy status at time of SARS-CoV-2 infection was confirmed through review of medical records.

**
https://covid.cdc.gov/covid-data-tracker/#variant-proportions

†† The total number of SARS-CoV-2 infections during pregnancy by period was obtained from MSDH COVID-19 case surveillance data. Pregnancy status for SARS-CoV-2 infections is ascertained by a pregnancy field on the case report form. The proportion of cases with known pregnancy status among females aged 10–49 years in Mississippi was higher at the beginning of the pandemic (March 2020–June 2020) when case counts were lower and decreased as case counts increased (July 2020–October 2021). However, the proportion of cases with known pregnancy status has remained relatively stable (approximately 17%) since July 2020.

§§
https://www.cdc.gov/nchs/data/statab/techap99.pdf

¶¶ 45 C.F.R. part 46, 21 C.F.R. part 56; 42 U.S.C. Sect. 241(d); 5 U.S.C. Sect. 552a; 44 U.S.C. Sect. 3501 et seq.

†††
https://www.cdc.gov/reproductivehealth/maternal-mortality/erase-mm/index.html

§§§
https://msdh.ms.gov/msdhsite/_static/31,0,299,359.html

¶¶¶ In Mississippi, during March 1, 2020–October 6, 2021, a total of 163,975 SARS-CoV-2 infections among females aged 10–49 years were reported, and 413 COVID-19–associated deaths occurred (2.5 deaths per 1,000 SARS-CoV-2 infections). During the pre-Delta period, 219 COVID-19–associated deaths occurred (2.1 deaths per 1,000 SARS-CoV-2 infections among females aged 10–49 years; 95% CI = 1.8–2.3); during the period of Delta predominance, 194 COVID-19–associated deaths occurred (3.3 deaths per 1,000 SARS-CoV-2 infections among females aged 10–49 years; 95% CI = 2.9–3.8).

**** https://covid.cdc.gov/covid-data-tracker/#vaccinations-pregnant-women (Accessed November 15, 2021).

†††† https://www.cdc.gov/vaccines/imz-managers/coverage/covidvaxview/interactive.html (Accessed November 15, 2021).

## References

[1] Zambrano LD, Ellington S, Strid P, ; CDC COVID-19 Response Pregnancy and Infant Linked Outcomes Team. Update: characteristics of symptomatic women of reproductive age with laboratory-confirmed SARS-CoV-2 infection by pregnancy status—United States, January 22–October 3, 2020. MMWR Morb Mortal Wkly Rep 2020;69:1641–7. 10.15585/mmwr.mm6944e3 33151921 PMC7643892

[2] Allotey J, Stallings E, Bonet M, ; PregCOV-19 Living Systematic Review Consortium. Clinical manifestations, risk factors, and maternal and perinatal outcomes of coronavirus disease 2019 in pregnancy: living systematic review and meta-analysis. BMJ 2020;370:m3320. 10.1136/bmj.m3320 32873575 PMC7459193

[3] Catalano A, Davis NL, Petersen EE, Pregnant? Validity of the pregnancy checkbox on death certificates in four states, and characteristics associated with pregnancy checkbox errors. Am J Obstet Gynecol 2020;222:269.e1–8. 10.1016/j.ajog.2019.10.005 31639369 PMC7056489

[4] Manning SE, Bennett A, Ellington S, Sensitivity of pregnancy field on the COVID-19 case report form among pregnancies completed through December 31, 2020: Illinois and Tennessee. Matern Child Health J 2021. Epub November 10, 2021. 10.1007/s10995-021-03263-8 PMC8580361 34761313

